# Current Treatments of Bruxism

**DOI:** 10.1007/s11940-016-0396-3

**Published:** 2016-02-20

**Authors:** Marc Guaita, Birgit Högl

**Affiliations:** Department of Neurology, Medical University of Innsbruck, Anichstrasse 35, 6020 Innsbruck, Austria

**Keywords:** Bruxism, Teeth grinding, Polysomnography, Treatment, Intraoral devices, Medications

## Abstract

Despite numerous case reports, the evidence for treatment of bruxism is still low. Different treatment modalities (behavioral techniques, intraoral devices, medications, and contingent electrical stimulation) have been applied. A clinical evaluation is needed to differentiate between awake bruxism and sleep bruxism and rule out any medical disorder or medication that could be behind its appearance (secondary bruxism). A polysomnography is required only in a few cases of sleep bruxism, mostly when sleep comorbidities are present. Counselling with regard to sleep hygiene, sleep habit modification, and relaxation techniques has been suggested as the first step in the therapeutic intervention, and is generally considered not harmful, despite low evidence of any efficacy. Occlusal splints are successful in the prevention of dental damage and grinding sounds associated with sleep bruxism, but their effects on reducing bruxism electromyographic (EMG) events are transient. In patients with psychiatric and sleep comorbidities, the acute use of clonazepam at night has been reported to improve sleep bruxism, but in the absence of double-blind randomized trials, its use in general clinical practice cannot be recommended. Severe secondary bruxism interfering with speaking, chewing, or swallowing has been reported in patients with neurological disorders such as in cranial dystonia; in these patients, injections of botulinum toxin in the masticatory muscles may decrease bruxism for up to 1–5 months and improve pain and mandibular functions. Long-term studies in larger and better specified samples of patients with bruxism, comparing the effects of different therapeutic modalities on bruxism EMG activity, progression of dental wear, and orofacial pain are current gaps of knowledge and preclude the development of severity-based treatment guidelines.

## Introduction

Bruxism is an oral motor condition which has raised interest in dental, sleep, and neurological research in the last 10 years, with more than 1200 articles published in MEDLINE including 151 review papers. Studies about prevalence, risk factors, pathophysiology, and current and new experimental therapies had important repercussions on treatment decisions.

Recently, an international expert commission has redefined bruxism as repetitive jaw muscle activity characterized by clenching or grinding of the teeth and/or by bracing or thrusting of the mandible that can occur during wakefulness (i.e., awake bruxism) or during sleep (i.e., sleep bruxism) [1••]. Awake bruxism is usually seen as a jaw clenching habit that appears in response to stress and anxiety states [2], while sleep bruxism represents a sleep related rhythmic masticatory activity generally associated with arousals (from sleep) [3, 4]. Both awake and sleep bruxism are sub classified into either *primary*, not related to any other medical condition, or *secondary*, associated to neurological disorders or considered an adverse effect of drugs [5–8].

Based on face-to-face questionnaires and telephone interviews, the prevalence of sleep bruxism (i.e., teeth grinding episodes during sleep) has been estimated around 8 % of the adult population and gradually decreases with aging [9, 10]. When both questionnaires and polysomnographic (PSG) criteria are considered, the prevalence is slightly lower with 5.5 %, as indicated by Maluly et al. in a sample of 1042 individuals [11••]. Regarding awake bruxism, around 20 % of the general population report awareness of pressing their teeth together, with predominance in females [12]. There is no available data in relation to prevalence of secondary bruxism, and most of the literature derives from cases reports.

Bruxism can lead to dental wear, jaw muscle pain and fatigue, and temporal headaches, and in some severe forms can compromise oral functions such as chewing, speaking, and swallowing [13]. However, no direct relationship has been observed between the type of bruxism, the severity, and the presence of additional clinical signs and symptoms. In fact, it has been reported that patients with frequent sleep bruxism are less prone to complain about fatigue and pain in the masticatory muscles than the patients with fewer masticatory events per night [14].

Awake bruxism is usually identified by the patient, but there are no objective methods that reliably quantify this behavior. In contrast, the American Academy of Sleep Disorders has proposed both clinical and PSG criteria for diagnosing sleep bruxism [15]. These advances in its measurement have helped to launch several randomized clinical trials (RCT) for evaluating the effects of different therapies on reducing the masticatory activity during sleep.

The aim of this article is to review the therapeutic approaches for treating bruxism and clarify the current evidence.

## Methods

A literature search was performed using MEDLINE/PubMed database, combining the terms “bruxism” OR “teeth grinding” OR “clenching” AND “treatment” as the first step in the research strategy. All abstracts in English were screened. We first selected RCT performed in adults older than 18 years with a clinical or PSG diagnosis of bruxism (awake bruxism and/or sleep bruxism), independently if they had or not comorbid sleep, neurological, or psychiatric disorders. Nonrandomized comparative studies, pre-treatment/post-treatment studies, cohort studies, and case reports were considered in case of scarce or lacking RCT.

## Primary bruxism

Effective management of primary awake bruxism is challenging. Since increased anxiety levels and somatization symptoms have been reported in these patients [16, 17], interventions such as counselling about triggers, habits modification, relaxation therapy, or biofeedback have been suggested to be appropriate [18]. However, no RCT exist to support these approaches.

In contrast to awake bruxism, several RCT have been performed to investigate the efficacy of different treatments in the reduction of bruxism activity during sleep. Most of them showed heterogeneous results. The treatments evaluated are as follows: (1) sleep hygiene measures combined with relaxation techniques, (2) splint therapy, (3) pharmacological therapy, and (4) contingent electrical stimulation. For further details, see Table 1.

**Table 1:** Primary sleep bruxism: overview of randomized clinical trials

Citation	Study type	Group	Intervention	Duration	Results
**Splint therapy**					
Dubé et al. [19]	Crossover RCT	9 definitive sleep bruxism	Occlusal splint Palatal splint	2 weeks	Both occlusal splints and palatal splints reduced rhythmic masticatory activity during the PSG.
Van der Zaag et al. [20]	Parallel RCT	21 probable sleep bruxism	Occlusal splint Palatal splint	4 weeks	Neither occlusal splints nor palatal splints reduced rhythmic masticatory activity during the PSG.
Harada et al. [21]	Crossover RCT^a^	16 probable sleep bruxism	Occlusal splint Palatal splint	6 weeks	Both occlusal splints and palatal splints reduced nocturnal masseter activity on portable EMG the night after the insertion of splints, but not after 2, 4, or 6 weeks of therapy.
Landry et al. [22]	Crossover RCT	14 definitive sleep bruxism	Occlusal splint Mandibular advancement device in 3 grades of protusion: • No protrusion • At 40 % • At >75 %	Irregular	Both occlusal splints and mandibular advancement devices reduced rhythmic masticatory activity during the PSG. Higher advancement with mandibular advancement devices was associated with larger decrease on rhythmic masticatory activity.
Landry-Schönbeck et al. [23]	Crossover RCT	12 definitive sleep bruxism	Occlusal splint Mandibular advancement device in 2 grades of protusion: • At 25 % • At 75 %	2 weeks	Mandibular advancement devices reduced rhythmic masticatory activity during the PSG. Occlusal splint tended to reduce rhythmic masticatory activity during the PSG (n.s.)
Arima et al. [24]	Crossover RCT^a^	11 possible sleep bruxism	Occlusal splint Mandibular advancement device in neutral position, in 2 conditions: • Not fix • Fix	1 week	Both occlusal splints and mandibular advancement devices reduced nocturnal masseter EMG activity on portable EMG. No differences between devices.
Madani et al. [25]	Single-blind, parallel RCT	20 definitive sleep bruxism	Occlusal splint Gabapentin 300 mg	8 weeks	Both occlusal splints and gabapentin reduced rhythmic masticatory activity during the PSG.
**Pharmacological therapies**					
Mohamed et al. [26]	Double-blind, crossover RCT^a^	10 probable sleep bruxism	Amitriptyline 25 mg Placebo	1 night	Amitriptyline did not reduce nocturnal masseteric EMG activity on portable EMG.
Lobbezzo et al. [27]	Double-blind, crossover, RCT^a^	10 definitive sleep bruxism	Levodopa/benserazide 100/25 mg Placebo	1 night	Levodopa reduced rhythmic masticatory activity during the PSG in 7 of 10 patients.
Lavigne et al. [28]	Double-blind, crossover RCT	7 definitive sleep bruxism	Bromocriptine 7.5 mg Placebo	2 weeks	Bromocriptine did not reduce rhythmic masticatory activity during the PSG.
Huynh et al. [29]	Crossover RCT^a^	25 definitive sleep bruxism	Study 1 (*N* = 10) Propranolol 120 mg Placebo Study 2 (*N* = 16) Clonidine 0.3 mg Placebo	1 night	Propranolol did not reduce rhythmic masticatory activity during the PSG. Clonidine decreased rhythmic masticatory activity during the PSG by > 60 %.
Shim et al. [30••]	Randomized, parallel, before-after study^a^	20 probable sleep bruxism	Botulinum toxin type A in masseters Botulinum toxin type A in masseters + temporalis	4 weeks	Botulinum toxin type A did not reduce rhythmic masticatory activity during PSG, but decreased the strength of contraction in the injected muscles. No differences between injecting 2 (masseters) or 4 (masseters + temporalis) muscles. 9/20 patients reported decreased teeth grinding after the therapy. 50 % reported improvement of morning jaw stiffness.
Lee et al. [31]	Double-blind, parallel, RCT^a^	12 possible sleep bruxism	Botulinum toxin type A Saline	12 weeks	Botulinum toxin type A did not reduce nocturnal masticatory activity on portable EMG, but decreased the strength of contraction in the injected muscles. Both botulinum toxin and saline injections improved subjective symptoms of sleep bruxism.
**Contingent electrical stimulation**					
Jadidi et al. [32]	Double-blind, parallel RCT^a^	11 probable sleep bruxism with myofascial pain	Contingent electrical stimulation Placebo	6 weeks	Contingent electrical stimulation reduced by 52 % nocturnal temporalis muscle activity on portable EMG during active therapy. No changes in self-reported muscle pain and tenderness were observed.
Conti et al. [33]	Single-blind, parallel RCT	15 probable sleep bruxism with myofascial pain	Contingent electrical stimulation Placebo	3 weeks	Contingent electrical stimulation reduced nocturnal temporalis muscle activity on portable EMG during active therapy. No changes were found in the placebo group. Contingent electrical stimulation did not influence perceived pressure pain thresholds or pain intensity.

The initial diagnosis of sleep bruxism is based on the diagnostic grading system proposed by Lobbezzo et al. [1••]: possible SB, based on questionnaires or the anamnestic part of the clinical history; probable SB, based on questionnaire and clinical examination; and definitive SB, based on questionnaires, clinical examination, and confirmed by PSG or portable EMG or audio-video recording. Duration makes reference to treatment duration

*EMG* electromyography, *PSG* polysomnography, *RCT* randomized clinical trials, *n.s*. not significant

^a^Randomization methods not further specified

### Sleep hygiene measures combined with relaxation techniques

The treatment of sleep bruxism usually begins with counselling of the patient with regard to sleep hygiene. This includes stop smoking and drinking of coffee or alcohol at night, limit the physical or mental activity before going bed, and ensure good bedroom conditions (quiet and dark). There is only one RCT that evaluated the effect of 4 weeks of sleep hygiene measures combined with relaxation techniques on sleep bruxism (as shown by PSG), but failed to find significant differences in comparison to baseline [34•]. Besides this negative result and the lack of evidence with other behavioral approaches, it is still reasonable to recommend good sleep hygiene in clinical practice, especially considering that alcohol, tobacco, and coffee consumption are risk factors for sleep bruxism [10, 35•] and that sensitivity to stress is commonly reported by the patients [36].

### Splint therapy

Occlusal splints have been considered as the first-line strategy for preventing dental grinding noise and tooth wear in primary sleep bruxism [37]. In general, the design of the device is simple, covers the whole maxillary or mandibular dental arch, and is well tolerated by the patient. However, its efficacy reducing the number of masticatory episodes per hour of sleep seems to be transient, with a maximal effect observed during the first 2 weeks [19], and returning to baseline after longer periods of use [20, 21, 24]. Only one study have compared occlusal splints versus a medication doses gabapentin, and found that both treatments reduced similarly the muscle activity associated with sleep bruxism after 2 month of therapy [25].

Albeit rare, occlusal splints may worsen respiration during sleep in patients with obstructive sleep apnea (OSA) and thus, special care should be taken when treating sleep bruxism in this population [38]. In the absence of larger studies that confirm this adverse effect, a mandibular advancement device might be an alternative treatment in patients with concomitant OSA and sleep bruxism, because it decreases both the number of sleep respiratory events in OSA and the sleep-related masticatory activity in sleep bruxers [22, 23, 39••, 40]. Again, to generalize this indication, more research needs to be done.

### Pharmacological therapy

Most of the drugs investigated for treating sleep bruxism were used in experimental studies of small sample size and in which the effects were solely evaluated after very short treatment periods using the medication. Mohamed et al. reported the first RCT evaluating amitriptyline (used during 7 days) in a group of patients with sleep bruxism and temporomandibular disorder symptoms, and found no changes in pain reports and in the nocturnal masseteric muscle activities with the therapy [26]. In contrast, Lobbezzo et al. evaluated the acute effect of levodopa in 10 severe sleep bruxers and found a decrease in the number of sleep-related masticatory events in 7 of them when compared to placebo. However, given the unknown clinical relevance and the lack of further research supporting its use, levodopa is not considered as a treatment for sleep bruxism [27]. Other pharmacological therapies such as bromocriptine and propranolol have also been investigated, but again failed to show positive results [28, 29].

On the other side, an experimental one-night treatment with clonidine, an α_2_ adrenergic agonist used for treating hypertension, attention-deficit hyperactivity disorder in children, and for acute alcohol or substanse withdrawal syndrome, has demonstrated to reduce bruxism activity by 60 %, but with significant adverse effects such as morning hypotension, REM sleep suppression, and dry mouth [29]. In patients with psychiatric and sleep comorbidities, the acute use of clonazepam has been reported to improve sleep bruxism activity together with the general quality of sleep, as suggested by Saletu et al. [41, 42]. However, these results are based on single-blind nonrandomized trials. To finally recommend both clonidine and clonazepam in the clinical routine, high-quality double-blinded RCT with larger sample size, different adjustable doses and larger duration, systematic assessment of adverse events, and perhaps also withdrawal studies are needed.

Recently, some studies have evaluated the efficacy of botulinum toxin type A injections into the masticatory muscles for treating sleep bruxism. Based on PSG, Shim et al. found that the amplitude of the muscle contraction during bruxism events was reduced after 4 weeks of injection, but with no changes in the rhythm or number of bruxism episodes per hour of sleep [30••]. Lee et al. reported similar results in an ambulatory EMG-based study over 8 to 12 weeks after botulinum toxin injection; however, no differences were observed in the relief of teeth grinding sounds and morning jaw stiffness in comparison to saline injections at any time of the study [31]. More RCT comparing the effect of botulinum toxin, saline injections, and dry needling are needed to clarify if botulinum toxin is useful in the management of sleep bruxism with and without orofacial pain.

### Contingent electrical stimulation

In the last years, contingent electrical stimulation (CES) has reappeared in an attempt to reduce the masticatory muscle activity associated to sleep bruxism. The rationale for CES consists in the inhibition of the masticatory muscles responsible of bruxism, applying a low-level electrical stimulation on the muscles when they become active, i.e. during the bruxism episode. Two experimental studies have applied CES in patients with signs and symptoms of sleep bruxism and myofascial pain, and found a reduction of the EMG episodes per hour of sleep while using CES, but with no changes in pain and muscle tension scores [32, 33]. Raphael et al. confirmed these results in a group of women with sleep bruxism and myofascial pain, but in addition showed that the efficacy of CES in reducing nocturnal bruxism events was confined to active periods and not after discontinuing the device [43]. Even if these results are promising, the unknown effect on tooth wear and the low impact on pain symptoms may limit the generalization of its use. Additional studies evaluating long-term treatment periods, the impact of contingent versus sustained electrical stimulation, and the combination with other therapies are still needed.

## Bruxism associated with neurological disorders

Bruxism has been documented in the context of some neurological disorders, mostly in form of case reports. Clinically, an acute onset of severe teeth grinding that occurs mainly during wakefulness and that cannot be prevented voluntarily should alert the clinician about the secondary etiology of bruxism. Moreover, it usually accompanies typical hallmarks of the underlying disease. For example, some patients with cranial and cervical dystonia show severe awake teeth grinding in combination with dystonic/dyskinetic movements in other parts of the face [44, 45]; in two patients with drug-resistant temporal lobe epilepsy, long-lasting teeth grinding episodes were the main features of the seizures, and usually preceded other typical signs such as the loss of consciousness and the limbs automatisms [46•, 47]; and in three women suffering from Huntigton’s disease, Tan et al. reported a persistent awake teeth grinding that worsened with the progression of the disease [13].

The management of bruxism associated with neurological disorders focuses on improvement of chewing, speaking, swallowing, and feeding, which are severely compromised, and to relieve orofacial pain symptoms. However, no general rules can be applied because placebo-controlled studies are lacking, so a case-by-case approach is suggested. Conservative measures such as occlusal splints and the use of drugs for treating the underlying disease may help the patient to control his bruxism; for example, a single case report in a patient with multiple system atrophy and constant awake teeth grinding claimed a marked improvement 1 week after starting levodopa-carbidopa, while similar results were documented after using galantamine in a patient with Alzheimer disease and awake bruxism [48, 49]. Injections of botulinum toxin in the masticatory muscles have been used in two case series of patients with severe bruxism associated with cranial dystonia and other movement disorders [44, 50]. In these studies, the therapy rendered the patients free of bruxism for up to 1–5 months and markedly improved pain reports and mandibular functions. Botulinum toxin was also effective in the resolution of bruxism that appeared during the recovering from coma in four patients with anoxic or traumatic brain injury [51–54]. Finally, surgical treatment of temporal lobe epilepsy resolved the occurrence of ictal bruxism in two reported cases [46•, 47]. It should be mentioned that intraoral devices should be used with caution in epilepsy patients as splints can break during seizures and obstruct the airway. A summary of the diseases with concomitant bruxism and the therapeutic approach is shown in Table 2.

**Table 2:** Bruxism associated with neurological disorders: characteristics and treatment outcomes

Disorder	Citation	Characteristics of bruxism	Treatment	Outcome
**Cranial and cervical dystonia**	Watts et al. [44] Tan et al. [45] Tan et al. [50] Pedemonte et al. [51]	Teeth grinding occurring during wakefulness and less frequently during sleep that accompanied other dystonic/dyskinetic movements. In some cases, orofacial functions such speaking, chewing, feeding and swallowing were impaired.	Botulinum toxin in masseters solely or in combination with temporalis and/or suprahyoid muscles^a^	Reduction of bruxism. Duration of response 13–19 weeks.
**Neurodegenerative disorders**				
Huntington’s disease	Tan et al. [13]	Awake teeth grinding reported by patients and familiars that worsens with the evolution of the disease.	Botulinum toxin in masseters^a^	Reduction of bruxism. Duration of response 8–17 weeks
Multiple system atrophy	Wali et al. [48]	Awake teeth grinding reported by patients and familiars, which disappeared during sleep.	Levodopa-carbidopa	Attenuation of bruxism
Alzheimer’s disease	Lai et al. [49]	Awake teeth grinding that disappeared while eating or speaking.	Galantamine	Reduction of bruxism
**Epilepsy**	Guaita et al. [46•] Meletti et al. [47]	Awake and sleep teeth grinding occurring during seizures, confirmed by video-EEG.	Temporal lobectomy	Disappearance of bruxism, confirmed by PSG in [46•]
**Anoxic/traumatic brain injury**	Van Zandijcke et al. [54] Ivanhoe et al. [53] El Maaytah et al. [52]	Sudden and continuous teeth grinding during the recovering of coma.	Botulinum toxin type A	Improvement of bruxism
**Stroke**	Tan et al. [55] Yi et al. [56]	Acute onset of awake teeth grinding, which improved during sleep.	Botulinum toxin type A [55] Metoclopramide [56]	Improvement of bruxism

*EEG*, electroencephalography, *PSG* polysomnography

^a^No specification of type of botulinum toxin

## Bruxism as an adverse effect of medications

A large number of clinical case reports have described the appearance of bruxism as an adverse effect of medications. Generally speaking, mostly middle-aged women with a wide spectrum of psychiatric disorders developed awake and/or sleep bruxism few weeks after starting the medicament. Patients or bed partners reported that bruxism augmented or decreased in accordance with dose manipulation and that in some cases definitely disappeared with drug withdrawal. Second-generation antidepressants have been the more cited drugs prone to cause (or exacerbate in some cases) bruxism, further followed by antipsychotics or bupropion. The list of medicaments associated with bruxism is shown in Table 3.

**Table 3:** Bruxism as an adverse effect of medication

Medication	Citation
**Selective serotonin reuptake inhibitors**	
Citalopram	[57]
Escitalopram	[58–61]
Fluoxetine	[62–65]
Paroxetine	[63, 66–70]
Sertraline	[63, 71]
**Serotonin and noradrenaline reuptake inhibitors**	
Duloxetine	[72]
Venlafaxine	[59, 73–78]
**Dopamine and noradrenaline reuptake inhibitors**	[79]
**Antipsychotics**	[80, 81]

Regarding the clinical management, some authors have suggested to “wait and see” for 1 month because spontaneous remission may occur [62]. If the problem persists, a reduction of dose or change of medication may alleviate or even resolve the problem [57–59, 73, 79], although a definitive cease might be expected with drug withdrawal [58, 63, 66, 72, 74, 75, 79, 81]. In those cases in which the psychiatric symptoms have been successfully improved with the current medication and no changes are planned, some authors have co-prescribed buspirone [57, 64, 71, 72, 75, 76], tandospirone [68], or gabapentine [65, 69, 77], reporting a complete cease of bruxism. However, caution mast be taking with the generalization of these findings, since case reports cannot establish cause-effect relationships and are susceptible to publication bias and over-interpretation. Based on these premises, costs and benefits of the drug manipulation should be considered individually and always performed by physicians. Moreover, polysomnographic and placebo-controlled studies evaluating the effects of antidepressants and other drugs on the occurrence of bruxism-like behavior require further research.

## Conclusion

In the absence of a causal treatment, the management of bruxism focuses to prevent progression of dental wear, reduce teeth grinding sounds, and improve muscle discomfort and mandibular dysfunction in the most severe cases. Counselling and behavioral strategies, splint therapy, medications, and contingent electrical stimulation have shown heterogeneous results in resolving the EMG events associated with sleep bruxism, and most of the RCT did not evaluate the effects on other symptoms such as pain or tooth wear progression. Long-term studies with a wide severity spectrum of sleep bruxism patients, and comparing the effect of different treatments should be performed to elucidate the importance of each intervention in the resolution of the signs and symptoms commonly referred by the patients. The choice of not treating bruxism must also be further explored, at least in asymptomatic patients with only mild dental wear. Even more must be done to successfully treat awake bruxism, in which RCTs are still lacking.

## Abbreviations

CES: Contingent electrical stimulation
EMG: Electromyography
OSA: Obstructive sleep apnea
PSG: Polysomnography
RCT: Randomized clinical trial
